# Novel Approaches with HIF-2α Targeted Therapies in Metastatic Renal Cell Carcinoma

**DOI:** 10.3390/cancers16030601

**Published:** 2024-01-31

**Authors:** Charles B. Nguyen, Eugene Oh, Piroz Bahar, Ulka N. Vaishampayan, Tobias Else, Ajjai S. Alva

**Affiliations:** 1 Rogel Comprehensive Cancer Center, University of Michigan, Ann Arbor, MI 48109, USA; vaishamu@med.umich.edu (U.N.V.); telse@med.umich.edu (T.E.); ajjai@med.umich.edu (A.S.A.); 2 Division of Hematology and Oncology, Department of Internal Medicine, University of Michigan, Ann Arbor, MI 48109, USA; 3 University of Michigan Medical School, Ann Arbor, MI 48109, USA; euoh@med.umich.edu (E.O.); baharp@med.umich.edu (P.B.); 4 Division of Metabolism, Endocrinology and Diabetes, Department of Internal Medicine, University of Michigan, Ann Arbor, MI 48109, USA

**Keywords:** renal cell carcinoma, RCC, kidney cancer, belzutifan, HIF-2α targeting, VHL, von Hippel-Lindau

## Abstract

**Simple Summary:**

Belzutifan, a hypoxia-inducible factor-2 alpha (HIF-2α) inhibitor, has emerged as an exciting new treatment option not only for patients with Von Hippel-Lindau (VHL)-related renal cell carcinoma (RCC) but also for sporadic RCC. While initial clinical data are promising, potential resistance with HIF-2α inhibitors may occur with increased understanding of this class of therapy. Potential ways to further increase the antitumor activity of HIF-2α targeting include combination strategies with immune checkpoint inhibitors and other targeted agents as well as newer generation HIF-2α inhibitors that are currently under development.

**Abstract:**

Germline inactivation of the Von Hippel-Lindau (*VHL*) tumor suppressor is the defining hallmark in hereditary VHL disease and VHL-associated renal cell carcinoma (RCC). However, somatic *VHL* mutations are also observed in patients with sporadic RCC. Loss of function *VHL* mutations result in constitutive activation of hypoxia-inducible factor-2 alpha (HIF-2α), which leads to increased expression of HIF target genes that promote angiogenesis and tumor growth. As of 2023, belzutifan is currently the only approved HIF-2α inhibitor for both VHL-associated and sporadic metastatic RCC (mRCC). However, there is potential for resistance with HIF-2α inhibitors which warrants novel HIF-2α-targeting strategies. In this review, we discuss the potential resistance mechanisms with belzutifan and current clinical trials evaluating novel combinations of belzutifan with other targeted therapies and immune checkpoint inhibitors which may enhance the efficacy of HIF-2α targeting. Lastly, we also discuss newer generation HIF-2α inhibitors that are currently under early investigation and outline future directions and challenges with HIF-2α inhibitors for mRCC.

## 1. Introduction

The treatment landscape for metastatic renal cell carcinoma (mRCC) has dramatically changed over the past few decades with the development of targeted therapies as well as immune checkpoint inhibitors (ICI). First-line therapy options for mRCC currently include dual ICI combinations with anti-CTLA-4 and anti-PD-1 inhibitors (e.g., ipilimumab and nivolumab) and tyrosine kinase inhibitors (TKIs), as well as the combination of ICIs with TKIs [1]. Despite these therapies, the estimated 5-year survival rate for patients with mRCC remains low at 15% [2]. Additionally, given the increasing prevalence of ICI-based therapies in the first-line and adjuvant settings in RCC, emerging prospective data have questioned the role of ICI rechallenge in patients with mRCC. In the recent CONTACT-03 trial, patients with mRCC who had disease progression on anti-PD-1-based therapies did not have improved clinical outcomes when immediately re-challenged with atezolizumab, a PD-L1 inhibitor, in combination with cabozantinib [3]. Thus, newer therapeutic targets for mRCC are urgently needed to address these unmet areas.

The *VHL* gene, found on chromosome 3p25, encodes for the VHL tumor suppressor protein that plays pivotal roles in hypoxia response pathway regulation [4]. Under normal oxygen conditions, VHL functions as a ubiquitin ligase that ubiquitinates the alpha subunit of the hypoxia-inducible factor (HIF) transcription factor, resulting in subsequent proteasomal degradation [5]. Inactivating *VHL* mutations lead to HIF stabilization and constitutive activation, resulting in enhanced expression of HIF-regulated genes which all contribute to angiogenesis and tumorigenesis [5,6,7]. Specifically, HIF induces the expression of *VEGF, PDGF-*β, and other angiogenic genes, leading to vascular permeability and endothelial cell differentiation [8]. In addition, HIF stimulates cellular growth by stabilizing and promoting the transcriptional activity of c-Myc which also leads to the enhanced expression of growth signaling genes such as *CCND1* [9]. Cyclin D1, encoded by *CCND1,* ultimately binds to cyclin-dependent kinase 4/6 (CDK4/6), resulting in downstream phosphorylation of the retinoblastoma (RB) tumor suppressor protein and subsequent release and nuclear localization of the E2F transcription factor that drives cell cycle progression [10]. *VHL* inactivation also leads to constitutive phosphorylation and activation of the c-MET receptor tyrosine kinase which interacts with the PI3K/AKT and RAS/MAPK growth pathways, among others [8,11]. HIF contributes to tumor immune escape by directly and indirectly (via c-MET activation) upregulating PD-L1, which inhibits cytotoxic T-cell activation and clonal expansion [12,13,14]. Additionally, HIF-2α is associated with diminished tumor-infiltrating lymphocytes and enhanced immunosuppressive IL-10 and TGF-β signaling [12]. Germline mutations in *VHL* lead to Von Hippel-Lindau (VHL) disease, an autosomal dominant inherited cancer syndrome. Patients with VHL disease have a near 70% lifetime risk of developing early-onset clear cell RCC (ccRCC), with an average age of RCC diagnosis of approximately 40 years [15]. Somatic mutations in *VHL* are found in about half of sporadic ccRCC cases [5]. Given the emerging data in RCC, the VHL pathway has been explored as a potential therapeutic target in both VHL-associated and sporadic RCC.

As of 2023, belzutifan is the only HIF-2α inhibitor that is Federal Drug Administration (FDA) approved for VHL-associated and sporadic RCC. In the phase 2 MK-6482-004 trial, patients with a confirmed pathogenic germline variant in *VHL* and localized RCC received an oral belzutifan dose of 120 milligrams (mg) daily [16]. At a median follow-up of 21.8 months, the study met the primary endpoint with an objective response rate (ORR) of 49% (95% CI 36–62). The progression-free survival (PFS) rate at 24 months was 96% (95% CI 87–99). Since the study also included patients with VHL-associated RCC and other VHL-associated tumors, responses were also observed in non-RCC lesions, including pancreatic neuroendocrine tumors (ORR 91%) and central nervous system hemangioblastomas (ORR 30%). The most common all-grade adverse events (AEs) with belzutifan in the study included anemia (90%), fatigue (66%), and headaches (41%). Based on the results of this study, the FDA approved belzutifan in August 2021 for the treatment of VHL-associated tumors including RCC [17]. Following this landmark approval for VHL-related tumors, belzutifan has been evaluated in other cancers including sporadic RCC. Ongoing clinical trials of belzutifan in patients with previously treated sporadic mRCC are underway. The LITESPARK-013 trial (NCT04489771) is a phase 2 study comparing two belzutifan doses (120 mg daily vs. 240 mg daily) in patients with sporadic mRCC who previously received up to three lines of prior therapy. The study showed that there was no significant difference in the primary endpoint of ORR as well as PFS and OS between either dose strengths [18]. However, the lower belzutifan dose of 120 mg was associated with less frequent rates of dose modification or discontinuation due to AEs and may represent the optimal starting dose [18]. The LITESPARK-005 trial (NCT04195750) is a randomized, phase 3 trial comparing belzutifan with everolimus, an mTOR inhibitor, in patients with sporadic mRCC who have received up to three lines of prior therapy. In the first interim analysis of 746 patients enrolled, belzutifan led to an improvement in one of the co-primary endpoints of PFS (HR 0.75, 95% CI 0.63–0.90, *p* < 0.001) and the secondary endpoint of ORR (22% vs. 3.5%, *p* < 0.00001), with more frequent complete response (CR) rates compared to everolimus [19]. These preliminary results from LITESPARK-005 led to the recent FDA approval of belzutifan in December 2023 for previously treated sporadic mRCC.

### Resistance Mechanisms of HIF-2α-Targeted Therapies

Despite the recent advances with belzutifan, the possibility of resistance to HIF-2α inhibition has emerged. While the MK-6482-004 trial, which led to the initial FDA approval of belzutifan in VHL-associated tumors, reported an ORR of 49%, two patients in the study had disease progression with belzutifan, raising the possibility of resistance [16]. Some potential resistance mechanisms to belzutifan and HIF-2α targeting have been proposed. In pre-clinical RCC models, including those with *VHL* alterations, resistance to PT2399 (a preclinical derivative of belzutifan) was associated with lower HIF-2α levels relative to sensitive cells (23% vs. 83%, *p* < 0.0001) but higher expression of *HIF1A* encoding for HIF-1α, which is another HIF that is activated during hypoxic stress [20,21]. RCC tumor xenografts developed resistance to PT2399 when treated for over 100 days, which may be mediated by a G323E mutation in *EPAS1* (endothelial PAS domain-containing protein 1) encoding for HIF-2α [20]. Similarly, in a companion analysis of patients enrolled in a phase 1 study of PT2385, another first-generation HIF-2α inhibitor, patients with disease progression had the *EPAS1* G323E mutation which prevented HIF-2 disassociation, thereby functioning as a “gatekeeper” mutation which may occur upon treatment or at baseline [22]. In the same study, a mutation in *TP53* was also identified at progression, indicating another potential resistance mechanism to HIF-2α-targeted therapies [22]. However, recent work has suggested that intact *TP53* status is not needed for HIF-2α inhibitor sensitivity [23]. Another proposed resistance mechanism with HIF-2α inhibitors includes alterations in the HIF-1/ARNT complex [20,24]. While the precise resistance mechanisms with belzutifan have not been reported, it is possible that similar mechanisms identified in the early generation of HIF-2α-targeted therapies may be involved. Overall, the potential for belzutifan resistance underscores the need for novel HIF-2α-targeting strategies and combinatorial approaches to further enhance efficacy (Table 1). 

## 2. Novel Belzutifan Combinations with Targeted Therapies

### 2.1. Belzutifan in Combination with Cabozantinib

Cabozantinib is an oral multi-targeted TKI with activity against VEGFR, c-MET, AXL, and RET that is currently approved for the treatment of metastatic RCC as a monotherapy or in combination with nivolumab [25,26]. HIF-2α notably increases *VEGF* expression and regulates angiogenesis [5,6]. The c-MET (*MET*) receptor tyrosine kinase is activated by the binding of hepatocyte growth factor (HGF), which results in the downstream cell signaling of various pathways involved in cellular growth and migration [27]. As discussed earlier, *VHL* mutations also lead to increased HGF/MET levels in RCC [28]. Thus, concurrent targeting of HIF-2α, VEGFR, and c-MET may be an efficacious approach.

The LITESPARK-003 study (NCT03634540) is an ongoing phase 2 study of belzutifan with cabozantinib in patients with metastatic ccRCC who are treatment-naïve (cohort 1) or have previously received up to two lines of systemic therapy including prior immunotherapy (cohort 2). In this study, patients receive oral belzutifan (120 mg daily) with oral cabozantinib (60 mg daily). In a preliminary analysis of 52 patients enrolled in cohort 2, the primary endpoint of ORR was 30.8% (95% CI 18.7–45.1), including one patient who had a CR and fifteen patients who had partial responses (PRs). Hypertension was the most frequent grade 3–4 AE in 27% of patients [29]. A subsequent update of the LITESPARK-003 study in October 2023 of 50 patients in cohort 1 and the 52 patients in cohort 2 showed an ORR of 70% (95% CI 55–82) and 31% (95% CI 19–45), respectively [30]. The median duration of response was 28.6 months and 31.5 months in cohorts 1 and 2. At a median follow-up of 24.3 and 40 months for cohorts 1 and 2, the reported median PFS was 30.3 months (95% CI 16-not reached [NR]) and 13.8 months (95% CI 9–19). The median OS was not reached in cohort 1 and 26.7 months (95% CI 20–41) in cohort 2 [30]. Taken together, a combination approach with belzutifan and cabozantinib may have synergistic activity by targeting multiple VHL-associated pathways in ccRCC.

### 2.2. Belzutifan in Combination with Lenvatinib

Lenvatinib is a multi-TKI targeting VEGFR1-3, c-Kit, FGFR1-4, PDGR-α, and RET that is currently approved in combination with pembrolizumab for first-line treatment of patients with mRCC and in combination with everolimus for patients who have previously received VEGF-targeted therapy [31,32,33]. The combination of belzutifan with lenvatinib was previously studied in the B5 arm of the phase 1/2 KEYMAKER-U03B study (NCT04626518). Preliminary data demonstrated an ORR of 50% (95% CI 29–71) in patients with mRCC who were previously treated with immunotherapy and VEGF-TKIs [34]. At a median follow-up after nearly 6 months, the median PFS was 11.2 months (95% CI 4-NR) with a six-month PFS rate of 55%. The most frequent AEs were anemia, fatigue, and hypertension at a frequency of 43% each [34]. The LITESPARK-011 trial (NCT04586231) is an ongoing randomized, phase 3 study of belzutifan with lenvatinib versus cabozantinib monotherapy in patients with mRCC who previously received immunotherapy [35]. The study has co-primary endpoints of PFS per blinded independent central review and overall survival (OS). 

### 2.3. Belzutifan in Combination with CDK4/6 Inhibitors

A previously discussed, loss-of-function *VHL* mutations lead to constitutive activation of HIF-2α, resulting in downstream upregulation of *CCND1* encoding for cyclin D1, a cell cycle regulator [7,36]. Cyclin D1 partners with CDK4 or CDK6 to drive cell-cycle progression through phosphorylation and inactivation of the RB tumor suppressor protein [37]. In pre-clinical models, treatment with PT2399 and a CDK4/6 inhibitor resulted in synergistic anti-tumor activity in *VHL*-deficient ccRCC cell cultures and xenografts [38]. Thus, there is potential preclinical rationale for the combination of belzutifan with CDK4/6 inhibitors in metastatic RCC.

Palbociclib is a selective CDK4/6 inhibitor that is currently approved for the treatment of hormone receptor-positive, HER2-negative, advanced breast cancer [39]. The LITESPARK-024 study (NCT05468697) is a randomized phase 1/2 trial comparing the combination of palbociclib with belzutifan versus belzutifan monotherapy in patients with metastatic ccRCC who have been treated with at least two lines of systemic therapy including prior immunotherapy and VEGF-targeted TKIs. The primary endpoint of the phase 2 portion is ORR with key secondary endpoints of OS, PFS, and safety. Abemaciclib is another CDK4/6 inhibitor that is approved for patients with advanced breast cancer and is currently being investigated in combination with belzutifan in a phase 1/1B study (NCT04627064). In this non-randomized study, patients with advanced ccRCC who have received at least one prior VEGF-TKI and one prior ICI will receive abemaciclib alone or in combination with belzutifan. The primary efficacy endpoint for both cohorts is ORR. 

## 3. Belzutifan Combinations with Immunotherapy

As discussed earlier, hypoxic conditions not only stimulate HIF signaling but also increase PD-L1 expression with cross-talk between both HIF and tumor immune pathways [13]. Increased HIF-2α levels are associated with diminished numbers of tumor-infiltrating CD8+ lymphocytes and promote stem cell factor (SCF) production, which in turn increases IL-10 and TGF-β secretion that ultimately contributes to an immunosuppressive tumor environment [12]. Thus, there are potential synergistic effects of combining HIF-2α inhibitors with ICIs.

### 3.1. Triplet Combinations with Belzutifan and Immunotherapy

In recent years, active clinical investigation in mRCC has moved from doublet-based strategies (e.g., dual ICI regimens and ICI-TKI combinations) to novel triplet therapy combinations. The COSMIC-313 trial was the first phase 3 study to evaluate a triplet-based combination in mRCC [40]. In this study, patients with untreated, intermediate/poor-risk mRCC were treated with the triplet combination of ipilimumab/nivolumab and cabozantinib versus ipilimumab/nivolumab and placebo. The study met the primary endpoint of PFS (HR 0.73, 95% CI 0.57–0.94, *p* = 0.01) in favor of ipilimumab/nivolumab and cabozantinib; however, the triplet regimen was notably associated with more frequent grade 3–4 AEs (79%) compared to the control group (59%) [40]. Additionally, no OS data have yet been reported, and there is a question as to why patients with poor-risk disease did not derive benefit from triplet therapy in the subgroup analysis compared to patients with intermediate-risk disease. Nonetheless, this trial was a novel study of triplet therapy in mRCC. 

An ongoing trial is evaluating the triplet combination of belzutifan with lenvatinib and pembrolizumab (NCT04736706). This phase 3 study will randomize patients with untreated metastatic ccRCC to either belzutifan/lenvatinib/pembrolizumab, belzutifan/lenvatinib with quavonlimab (anti-CTLA-4 ICI), or lenvatinib/pembrolizumab [41]. The co-primary endpoints for this trial are PFS and OS with key secondary endpoints of ORR and toxicity. Similar trials with the triplet combination of belzutifan with lenvatinib and pembrolizumab are also ongoing (NCT05899049 and NCT05030506). 

### 3.2. Belzutifan and Immunotherapy Combinations in the Adjuvant Setting

The data supporting the use of adjuvant treatment for localized RCC have been mixed. Two studies evaluating the role of adjuvant sunitinib (S-TRAC and ASSURE trials) demonstrated conflicting results regarding the primary endpoint of disease-free survival (DFS) in patients with high-risk, localized RCC, although potential differences in patient selection in both studies could have explained the contradictory outcomes [42,43]. In the current immunotherapy era, pembrolizumab is the only ICI approved for adjuvant therapy in patients with ccRCC who have a high risk of recurrence following resection based on the results of the KEYNOTE-564 trial. This study demonstrated an improvement in DFS with pembrolizumab compared to placebo [44]. Interestingly, trials of other ICI agents in the adjuvant setting have yielded negative results [45,46]. Nonetheless, building on the DFS benefit of adjuvant pembrolizumab, the LITESPARK-022 trial (NCT05239728) is an ongoing phase 3 study evaluating the combination of pembrolizumab and belzutifan versus pembrolizumab and placebo as adjuvant therapy in patients with ccRCC following nephrectomy or metastasectomy who are at high risk of disease recurrence [47]. The study’s primary endpoint is DFS with secondary endpoints including OS and safety. 

### 3.3. Belzutifan Combination with Anti-TIGIT Therapies

An emerging ICI target is a T cell immunoreceptor with immunoglobulin and an ITIM domain (TIGIT) which is involved in immune-mediated tumor recognition [48]. Early phase studies evaluating anti-TIGIT antibodies have demonstrated potential antitumor activity in a subset of patients with metastatic solid tumors [49,50,51]. The combination of vibostolimab, a novel anti-TIGIT agent, with pembrolizumab was investigated in a phase 1 study which showed an ORR of 26% in patients with advanced non-small cell lung cancer who have not received ICIs [52]. There is an ongoing phase 1b/2 study of a proprietary co-formulation of vibostolimab and pembrolizumab with the addition of belzutifan as a first-line therapy for patients with mRCC (NCT04626479). 

## 4. Other Novel HIF-Targeted Therapies in RCC

The clinical success of belzutifan has paved the way for other newer generation HIF-2α-targeted agents in the drug development pipeline. There are both pre-clinical and clinical-level agents differentiated from belzutifan that are currently in development for RCC, with some demonstrating early promise for improved therapeutic outcomes (Table 2).

### 4.1. DFF332

DFF332 is a small-molecule HIF-2α inhibitor that is being studied in a phase 1/1b study as a monotherapy and in combination with everolimus (an mTOR inhibitor) or spartalizumab (an anti-PD-1 ICI), plus taminadenant (an adenosine A2a receptor antagonist) in patients with mRCC and other tumors harboring HIF alterations (NCT04895748). The primary endpoints for this dose escalation and expansion study include safety and frequency of dose-limiting toxicities, with key secondary endpoints of ORR and PFS [53].

### 4.2. ARO-HIF2 (RNA Interference)

Targeted RNA silencing or interference of gene expression holds promise as a novel therapeutic mechanism. In pre-clinical models, novel small interfering RNA (siRNA) against HIF-2α such as ARO-HIF2 were shown to decrease HIF-2α levels and tumor volume in ccRCC tumorgraft models [54]. More recently, ARO-HIF2 is being evaluated in a phase 1 study of patients with previously treated mRCC (NCT04169711). This study has a primary endpoint of safety and secondary endpoints including tumor response. In an interim analysis of the first 23 patients enrolled, fatigue was the most frequent AE reported in 39% of patients [55]. Serious AEs were reported in three patients, including hypoxia, myocarditis, and neuropathy. Efficacy analysis showed a disease control rate (CR, PR, and stable disease) of 30% [55]. Correlative analysis of enrolled patients who underwent on-treatment biopsies showed that ARO-HIF2 led to reductions in HIF-2α mRNA and protein levels. Overall, this initial data showed proof-of-concept of RNA-based therapies targeting HIF-2α expression, although further clinical investigation is needed to elucidate its efficacy.

### 4.3. NKT-2152

NKT-2152 is a novel small-molecule HIF-2α inhibitor under investigation. In cell culture and mouse models, NKT-2152 was shown to interfere with HIF-2α degradation and disrupt the HIF-2α/HIF-1β complex, leading to decreased nuclear localization of HIF-2α and subsequent expression of HIF-2α target genes such as *VEGF-A*, *GLUT1*, and *CCND1* (cyclin D1) [56]. A phase 1/2 trial (NCT05119335) of NKT-2152 is currently ongoing in patients with previously treated mRCC with primary endpoints of identifying the recommended dose for expansion (phase 1 portion) and ORR (phase 2 portion). Another phase 2 study (NCT05935748) is investigating the combination of NKT-2152 with palbociclib, a CDK4/6 inhibitor, with or without sasanlimab, a novel anti-PD-1 ICI, in patients with previously treated mRCC. The primary endpoints of this study include the frequency of dose-limiting toxicities and ORR.

### 4.4. AB521

Another novel HIF-2α inhibitor identified is AB521, which inhibits HIF-2α binding to HIF-1β and therefore disrupts expression of HIF-2α target genes, which has been investigated in pre-clinical models [57]. In RCC tumor xenograft models, treatment with AB521 resulted in a dose-dependent reduction in tumor size [58]. Furthermore, the combination of AB521 with cabozantinib resulted in potential synergistic activity in pre-clinical models compared to either agent alone [59]. In a pharmacokinetic and pharmacodynamic phase 1 study, AB521 was shown to decrease circulating levels of erythropoietin (EPO) in a dose-dependent manner [60]. There is currently an ongoing phase 1 trial (NCT05536141) that is investigating the safety and efficacy of AB521 in patients with mRCC who have previously received anti-PD-1 or TKI therapy, as well as in patients with other advanced solid tumors. 

### 4.5. BPI-452080

BPI-452080 is a selective small-molecule HIF-2α inhibitor that disrupts HIF-2α heterodimerization with HIF-1β, leading to decreased transcription of downstream hypoxia-responsive genes (e.g., *GLUT1, CCND1*, and *CXCR4*) and VEGFA secretion in pre-clinical RCC models [61]. In RCC tumor xenograft models, oral treatment with BPI-452080 led to a dose-dependent reduction in tumor size [61]. As of early 2023, a phase 1 clinical trial of BPI-452080 (NCT05843305) is ongoing in China to evaluate the safety and efficacy in patients with mRCC (including VHL-associated mRCC) and other advanced solid tumors.

### 4.6. KD061

Ferroptosis is the process of cellular accumulation of cytotoxic iron-dependent lipid peroxides that results in programmed cell death, in a manner that is distinct from apoptosis and autophagy, and may be a potential target in mRCC [62]. KD061 is a recently identified molecule that targets iron-sulfur cluster assembly 2 (ISCA2), which is involved in ferroptosis and also decreases HIF-1/2α levels [63]. In pre-clinical RCC models, treatment with KD061 resulted in decreased tumor growth and HIF levels as well as induced ferroptosis [63]. Thus, dual targeting of HIF and ferroptotic pathways may overcome therapeutic resistance and further increase the efficacy of HIF-targeted inhibition. It will be interesting to see if KD061 will transition to first-in-human early-phase clinical studies in the future. 

## 5. Broadening the Use of HIF-2α-Targeted Therapies to Other Cancers

While HIF-2α clearly plays critical roles in ccRCC, there is emerging data that HIF-2α may also be implicated in other cancers such as breast, colorectal, liver, and prostate cancers [64,65,66,67]. A few prospective studies evaluating the efficacy of belzutifan in other cancers beyond mRCC are currently ongoing. MK-6482-015 is a phase 2 study (NCT04924075) evaluating the efficacy of belzutifan in patients with advanced pheochromocytoma/paraganglioma, neuroendocrine tumors of the pancreas, and gastrointestinal stromal tumors (GIST), among others. Belzutifan is also being studied in combination with pembrolizumab in patients with metastatic castration-resistant prostate cancer (NCT02861573) as well as in the neoadjuvant setting in combination with abiraterone acetate, prednisone, and leuprolide acetate in patients with regional node-positive prostate cancer (NCT05574712). 

## 6. Conclusions and Future Directions

HIF-2α targeting with belzutifan holds exciting promise and is a welcomed therapeutic addition to the evolving treatment landscape for patients with VHL-associated and sporadic mRCC. Since most first-line systemic therapy options for mRCC largely include immunotherapy-based combinations, HIF-2α inhibitors fulfill the need for newer therapies with novel mechanisms of action for patients who have previously been treated with ICIs, although clinical trials of HIF-2α inhibitors in the first-line setting are ongoing. As the treatment of mRCC now enters the era of HIF-2α-targeted therapies, an understanding and recognition of the potential resistance to HIF-2α inhibitors that may emerge with longer follow-up will be critical moving forward. Potential combinatorial strategies with HIF-2α agents and immunotherapy and other targeted therapies may further enhance the efficacy of HIF-2α inhibitors. Other newer generation HIF-2α inhibitors are also under early phase clinical investigation, which may lead to additional therapy options for patients with mRCC. It is currently unknown if newer generation HIF-targeted agents can overcome the *EPAS1* G323E resistance mutation. Future HIF-2α inhibitors should be designed to potentially target this “gatekeeper” G323E mutation and other mediators of resistance within the VHL/HIF pathway. An additional potential challenge that could emerge is determining the optimal sequencing of therapies, including HIF-2α inhibitors. Future prospective studies should evaluate if HIF-2α-targeted therapies and combinations have the greatest clinical benefit either in the first-line or subsequent settings, as well as investigate if HIF-2α inhibitors can be continued or re-challenged at progression. Currently, there are no predictive biomarkers in clinical practice to select therapies such as immune-based or TKI-based regimens for patients with mRCC. While *VHL* alterations are frequent in mRCC, not all patients with mRCC will harbor *VHL*-inactivating mutations [4,68]. Thus, an enhanced selection of patients with mRCC who have VHL pathway alterations or HIF-sensitizing mutations by incorporating biomarker analysis may be needed to identify the subgroup of patients that may or may not respond to HIF-2α inhibitors.

## Figures and Tables

**Table 1:** Currently active clinical trials of belzutifan in renal cell carcinoma (RCC) as of October 2023.

Trial Name/NCT Identifier	Phase	Treatment	Setting	Key Primary Endpoint(s)
Monotherapy trials				
NCT04846920	1	Belzutifan monotherapy (dose escalation)	Advanced refractory ccRCC	Advderse events; percentage of participants who discontinue or modify/interrupt treatment due to adverse event; DLTs
LITESPARK-013 (NCT04489771)	2	Belzutifan (120 mg versus 240 mg)	Advanced refractory ccRCC	ORR
LITESPARK-005 (NCT04195750)	3	Belzutifan monotherapy	Advanced refractory ccRCC	PFS and OS (co-primary)
Combinations with targeted therapies				
LITESPARK-024 (NCT05468697)	1/2	Belzutifan + Palbociclib	Advanced refractory ccRCC	Adverse events; DLTs; number of participants who discontinue treatment due to adverse event; ORR (phase 2)
NCT04627064	1/1B	Belzutifan + Abemaciclib	Advanced refractory ccRCC	Maximum tolerated dose and ORR
LITESPARK-003 (NCT03634540)	2	Belzutifan + Cabozantinib	Advanced refractory ccRCC	ORR
KEYMAKER-U03B (NCT04626518)	1/2	Belzutifan + Lenvatinib	Advanced refractory ccRCC	Adverse events; DLTs; number of participants who discontinue therapy due to adverse event; ORR
LITESPARK-011 (NCT04586231)	3	Belzutifan + Lenvatinib	Advanced refractory ccRCC	PFS and OS (co-primary)
Combinations with immunotherapy				
LITESPARK-022 (NCT05239728)	3	Belzutifan + Pembrolizumab	Adjuvant therapy	Disease-free survival
LITESPARK-012 (NCT04736706)	3	Belzutifan/Lenvatinib + Pembrolizumab or Quavonlimab	First-line in advanced ccRCC	PFS and OS (co-primary)
NCT05899049 (China)	3	Belzutifan + Pembrolizumab + Lenvatinib	First-line in advanced ccRCC	PFS and OS (co-primary)
NCT05030506 (China)	1	Belzutifan + Lenvatinib +/− Pembrolizumab	First-line in advanced ccRCC	Adverse events; DLTs; pharmacokinetic/pharmacodynamic profiles
NCT04626479	1/2	Belzutifan + Vibostolimab/Pembrolizumab	First-line in advanced ccRCC	Adverse events; DLTs; ORR

Abbreviations: ccRCC, clear cell renal cell carcinoma; DLT, dose-limiting toxicities; NCT, National Clinical Trial; ORR, objective response rate; OS, overall survival; PFS, progression-free survival

**Table 2:** Currently active clinical trials of newer generation HIF-2α inhibitors for mRCC as of October 2023.

Trial Name/NCT Identifier	Phase	Agent	Setting	Key Primary Endpoint(s)
Novel small molecule inhibitors				
NCT04895748	1/1b	DFF332 monotherapy DFF332 + Everolimus/Spartalizumab + Taminadenant	Advanced refractory ccRCC	Adverse events; DLTs; number of participants with dose interruptions/reductions; dose intensity for dose escalation/expansion
NCT05119335	1/2	NKT-2152	Advanced refractory ccRCC	DLTs; recommended dose for expansion in the dose escalation phase (phase 1); recommended dose for phase 2; ORR (phase 2)
NCT05935748	2	NKT-2152 + Palbociclib + Sasanlimab	Advanced refractory ccRCC	DLT and ORR
ARC-20 (NCT05536141)	1	AB521	Advanced refractory ccRCC	DLTs and adverse events
NCT05843305	1	BPI-452080	Advanced refractory ccRCC	Adverse events
RNA interference (RNAi)				
NCT04169711	1	ARO-HIF2	Advanced refractory ccRCC	Adverse events

Abbreviations: ccRCC, clear cell renal cell carcinoma; DLT, dose-limiting toxicities; NCT, National Clinical Trial; ORR, objective response rate
